# Selection of Mercury-Resistant PGPR Strains Using the BMRSI for Bioremediation Purposes

**DOI:** 10.3390/ijerph18189867

**Published:** 2021-09-18

**Authors:** Daniel González, Marina Robas, Agustín Probanza, Pedro A. Jiménez

**Affiliations:** Department of Pharmaceutical Science and Health, Montepríncipe Campus, CEU San Pablo University, Ctra. Boadilla del Monte Km 5.300, 28668 Boadilla del Monte, Spain; a.probanza@ceu.es (A.P.); pedro.jimenezgomez@ceu.es (P.A.J.)

**Keywords:** heavy metal pollution, bioremediation, PGPR, BMRSI, mercury

## Abstract

Heavy metal pollution of soil, particularly by mercury (Hg), is a problem that can seriously affect the environment and human health. For this reason, it is necessary to take steps to remediate these environments, prevent potential adverse effects, and restore these areas for subsequent use in agriculture, industry, ranching, and forestry. The present study has selected 40 bacterial strains from rhizosphere and bulk soil that grow naturally in high Hg-contaminated soils from the Almadén mining district in Ciudad Real, Spain. With the objective of evaluating the potential use of these strains in phyto-rhizoremediation, an evaluation and statistical analysis of their PGPR (Plant-Growth-Promoting Rhizobacteria) activity at different levels of Hg was carried out as the first condition of selection for their potential use in bioremediation. In addition, a Hg MBC (Maximum Bactericidal Concentration) was performed with the aim of selecting the strains with high Hg tolerance. Finally, strains with potential biotechnological use have been proposed according to the Bio-Mercury Remediation Suitability Index (BMRSI) criteria, which consider indole-3-acetic acid (IAA) production, acid 1- aminocyclopropane-1-carboxylic deaminase (ACCd) activity, phosphates solubilization, and siderophore production measured in the presence of Hg, as well as its MBC to Hg. The strains selected for further in vivo and in situ processes must reach at least an MBC (Hg) > 100 μg/mL and BMRSI ≥ 6.5.

## 1. Introduction 

Mercury (Hg) is an element with a high level of toxicity that poses a serious environmental threat. It is possible for Hg to enter the food chain and consequently affect human health, even at very low concentrations [1,2]. From a toxicological point of view, Hg is a toxic metal without any specific biological function. This element has a high potential for bioaccumulation and biomagnification due to the high solubility of Hg and methylmercury in fat and muscle tissue. An accumulation of Hg can result in pathologies of the central nervous system, such as Minamata’s Syndrome [3], as well as other health problems related to development, growth, and fertility [4].

On a global scale, anthropogenic emissions add approximately 2500 Mg of Hg to the atmosphere every year. In Europe, Lado et al. [5] developed a model of Hg distribution in the soil in 28 countries. According to their research, the average amount of Hg in European soil was around 40 μg kg^−1^. Soils in Northern Europe have a higher concentration of Hg than soils in the countries of Central and Southern Europe due to the fact that cold, wet weather promotes the accumulation of Hg in organic matter in soil [6]. Studies related to Hg distribution in soils adjacent to the Hg mines in the Almadén mining district reveal the presence of both high and extremely high levels of Hg (up to 8889 μg/g), while the concentration in sediment and water reaches levels of up to 16,000 μg/g and 11.2 μg/L, respectively [7].

In 2003, after more than 2000 years of activity, the mines closed due to a decrease in the demand for Hg, as well as changes in European regulations regarding this metal. With the aim of providing alternative uses for the Almadén soil, the scientific community has been working to develop strategies to mitigate the effects of Hg. Certain physicochemical methods have been developed that enable the elimination of this metal from soil, but the current trend is to use biological methods that are more environmentally friendly, based on biotechnological techniques such as bioremediation. This is the case with phyto-rhizoremediation, which involves the synergistic collaboration of plants and microorganisms for the purpose of remediating chemical compounds and pollutants from the environment [8]. An example of this activity is the use of plant growth-promoting rhizobacteria (PGPR) [9], which can be used in phyto-rhizoremediation aimed at the plant’s root in order to aid its physiological development, as well as direct activity aimed at the pollutant, while simultaneously increasing the effect of the plant itself on the pollutant. 

Hg tolerance and Hg resistance of microorganisms can contribute to the reduction and/or elimination of the different types of Hg in contaminated environments, which has led to increased interest in the selection of bacterial strains with biotechnological potential, as well as their use in bioremediation [10]. 

The Bio-Mercury Remediation Suitability Index (BMRSI) has proven to be a useful tool for evaluating the Hg bioremediation potential of the bacterial strains, since it takes into account not only the Hg resistance capability of the bacteria, but also their combined PGPR capacity. For this reason, the present study proposes a BMRSI analysis of the best forty bacterial strains obtained by Robas et al. [11] in the presence of Hg.

## 2. Materials and Methods

### 2.1. Bacteria Analyzed

This study was carried out with samples from the Almadén mining district in Ciudad Real, Spain. An experimental plot was used (*Plot M)*, as it has been classified as an area of high Hg contamination with concentrations of 1710 mg/kg Hg [12]. The plants used for the bacterial extraction from the rhizosphere were the following: *Rumex induratus* Boiss. and Reut., *Rumex bucephalophorus* L., *Avena sativa* L., *Medicago sativa* L., and *Vicia benghalensis* L., in addition to bulk soil. All the plant species were sampled in situ at *Plot M* during the spring season, looking for the maximum biological activity in that region. The plant samples were carried to the laboratory at 4 °C and processed before 24 h.

The bacteria selected for the study were isolated, characterized, identified, and selected as effective Hg remediators by measuring their capabilities as PGPR in the absence of Hg, by Robas et al. [11], as shown in Table 1. All the isolated strains were stored at −80 °C before their use in the present study.

### 2.2. Testing PGPR Activity

Each PGPR activity was tested according to the protocols described in the bibliography. These protocols were modified in an innovative way in order to test the PGPR capacity of the isolates in the presence of different concentrations of Hg. The objective was to validate this method of analyzing PGPR activity [11] in the presence of heavy metal by using the BMRSI.

The concentrations of Hg tested in each protocol were 80 μg/mL, 100 μg/mL, 120 μg/mL, and 140 μg/mL of Hg Cl_2_. 

To determine the production capacity of Indole-3-Acetic Acid (IAA) in vitro, a colorimetric technique with the reagent Van Urk Salkowski from the Salkowski method was used [13]. The isolated bacteria were grown in LB broth (Lennox) with the proposed protocol modification and incubated at 28 °C for 48 h with the IAA concentration measured at intervals of 12 h, 24 h, and 48 h. The results were quantified in μg/mL. 

To determine the ability of the strains to degrade acid 1- aminocyclopropane-1-carboxylic (ACC) through the activity of ACC deaminase, the protocol described by Glick [14] was followed and modified, as described above.

The siderophore production was determined by the use of Chrome Azurol S (CAS) agar described by Alexander and Zuberer [15] and modified by the addition of Hg.

The ability to solubilize inorganic phosphates was determined by the use of the protocol described by de Freitas et al. [16] and modified, as in the previous case.

### 2.3. Maximum Bactericidal Concentration of Hg (MBC)

To study the Hg MBC, the selected bacteria were seeded on Müller Hinton agar plates of the commercial brand Pronadisa^®^ (Eucast, 2017, Växjö, Sweden) following the protocol and criteria established by Robas et al. [11].

### 2.4. Bio-Mercury Remediation Suitability Index (BMRSI)

To evaluate the bio-mercury remediation potential of the strains, the BMRSI proposed by Robas et al. [11] was used. BMRSI measures the bioremediation potential of the strains by the inclusion of different PGPR activities and its Hg MBCs in one formula:

BMRSI = [IAA (µg/mL) + ACCd (1/0) + SID (cm) + PO_4_^3−^ (1/0)] + [MBC Hg (µg/mL)]

where: Presence = 1; Absence = 0.

### 2.5. Data Processing 

Using the results of the auxin production, descriptive statistical analyses were carried out using the SPSS v26.0 program (Version 26.0 IBM Corporation). The purpose of these analyses was to ensure that the modification of the protocols for this study provides statistical significance to the data obtained in the experiments. In order to determine which group of IAA production data based on the Hg concentrations tested would be subsequently included in the BMRSI, a statistical ANOVA analysis was performed at each of the intervals tested (12 h, 24 h, and 48 h). When a significance level of *p* < 0.05 was obtained, a post hoc analysis was then carried out using the Bonferroni test.

## 3. Results

The selected strains were subjected to more extensive tests in order to identify the best candidates for further uses in phyto-rhizoremediation based on their PGPR capabilities in the presence of Hg.

For this purpose, only the data obtained in the Hg tests were analyzed due to the fact that the final objective of this study was to analyze the remediation capability in the presence of Hg, as well as the choice of the best strains for use in the bioremediation of plots contaminated with this heavy metal.

**Figure 1:**
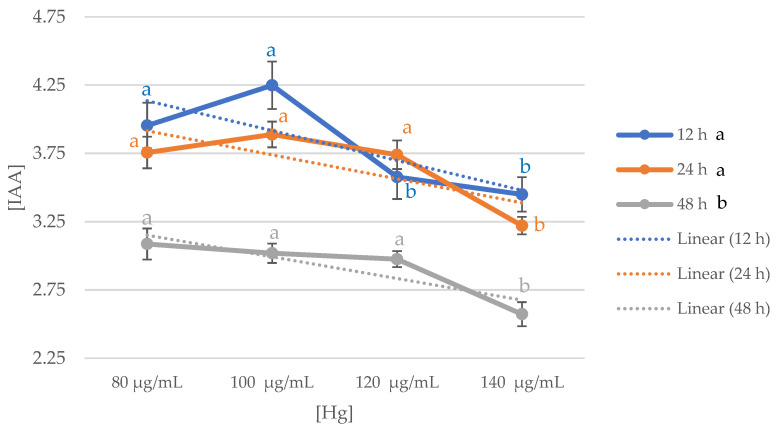
Average IAA production trend of the forty strains studied in relation to Hg concentrations at each of the measurement times. Letters a-b indicate the significance of *p* < 0.05, the black letters indicate significance differences among those grouped by hours; the blue letters indicate significance differences among the different concentrations of IAA measured at 12 h; orange letters indicate significance differences among different concentrations of IAA measured at 24 h; grey letters indicate significance differences among different concentrations of IAA measured at 48 h.

Figure 1 shows the trend of IAA production at different Hg concentrations over time (12 h, 24 h, and 48 h) in all the strains. Data measured at 12 h and 24 h were found to be significantly higher than those measured at 48 h (*p* < 0.05). By analyzing the mean values, it was found that during 12 h incubation period, the production of IAA was significantly higher at concentrations of 80 μg/mL and 100 μg/mL than at 120 μg/mL and 140 μg/mL of Hg (*p* < 0.05). However, in the incubation period of 24 h and 48 h, at concentrations of 80 μg/mL, 100 μg/mL, and 120 μg/mL, IAA production was significantly higher (*p* < 0.05 and *p* < 0.005, respectively), than at concentrations of 140 μg/mL Hg.

Therefore, it can be concluded that the average production value of the strains analyzed is obtained between 12 h and 24 h at concentrations between 80 μg/mL and 100 μg/mL. To select the range of data to be used later, values corresponding to the 12 h incubation period in mediums with a concentration of 100 μg/mL were used as a reference.

As shown in Table 2, only five strains (9, 48, 58, 122, and 173) exhibited ACCd activity. All of these showed activity up to concentrations of 100 μg/mL, and two of them (strains 9 and 58), up to 120 μg/mL of Hg.

Only three strains (50, 57, and 69-II) solubilize phosphates under the Hg conditions studied.

The production of siderophores was not included in Table 2 since no strain is safe to produce in the presence of Hg.

Regarding MBC, all strains resisted concentrations above 100 μg/mL. The minimum concentration resisted by the 40 strains was 140 μg/mL. A total of 55% of the strains tested resisted up to 140 μg/mL, but the other half had much higher resistance values. In the remaining 45%, we found nine strains that resisted up to 160 μg/mL, two up to 180 μg/mL, four up to 200 μg/mL, and three of them resisted up to 350 μg/mL.

Finally, after the analysis was carried out for each of the variables, the BMRSI was calculated using the data of the PGPR activity measured at 100 μg/mL in order to introduce the least possible variability and obtain uniform data from the sample.

Table 2 shows the integrated data of the PGPR and MBC activities of the 40 strains considered for evaluation using the BMRSI.

When the measurement is standardized at 100 μg/mL of Hg, it can be observed that the datum with greater weight in the calculation is the amount of IAA produced by each strain. Similarly, the production of siderophores for all the strains in the selected conditions is 0. Therefore, strains with high IAA production that exhibit other PGPR activity will have a higher BMRSI. Similarly, the taxonomic identification of the 40 selected strains can be observed in Table 2 [11].

**Figure 2:**
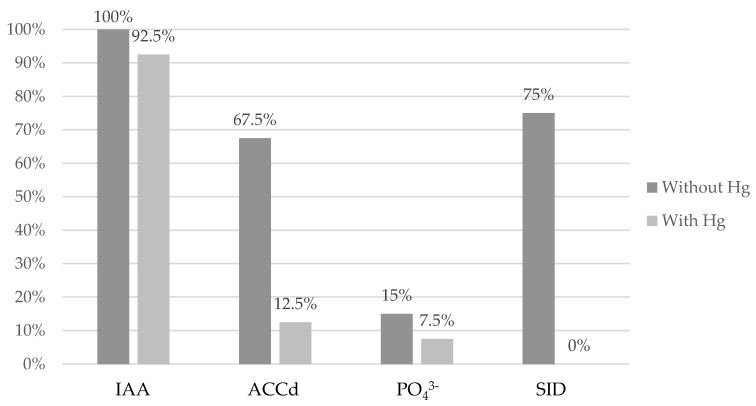
Percentage comparison of the data obtained by Robas et al. [11], contrasted with those obtained in the present study of the number of strains with PGPR activity at 0 μg/mL and 100 μg/mL of Hg. IAA: auxin producers; ACCd: ACC degraders; PO_4_^3−^: phosphate solubilizers; and SID: siderophore producers.

As such, a percentage comparison was made of the number of bacteria that exhibit each of the PGPR activities at 0 μg/mL of Hg obtained by Robas et al. [11], compared to those obtained using the selection criterion of 100 μg/mL of the present study, as shown in Figure 2. As can be seen in Figure 2, a reduction in the PGPR capacity of the bacteria under study occurs when these activities are analyzed in the presence of Hg.

## 4. Discussion

The fraction of soil surrounding the plant roots (rhizosphere) provides an environment that enables the growth of a large number of microorganisms [17]. Among these microorganisms, PGPRs have been shown to assist plant growth [18]. In addition, those that were able to support plants in their phytoremediation activity against heavy metals, including Hg [19,20,21,22,23], are of particular interest for the present study. In this research, we have selected and classified PGPR strains based on their quantified remediation potential using the BMRSI described by Robas et al. [11].

Most of the studies that have focused on the search for metallotolerant PGPR bacteria have generally been oriented toward specific bacterial genera, such as *Bacillus* [24], *Azotobacter* [25], or *Pseudomonas* [26], among others. The source of isolation is usually plants for agricultural use [27,28,29] since wild plants are rarely studied for isolation [19]. In the study herein, native plants from the Almadén mining district were used as a source of isolation, thereby maximizing the probability of selecting strains with potential biotechnological use due to the selective pressure of the heavy metal and the co-evolution/coadaptation with the plant.

Of all PGPR activities, the production of auxins, which regulate cell germination and elongation, as well as root formation, is important. However, as indicated by Mirza et al. [30], the production of IAA by PGPR bacteria may vary among species and strains, as well as among conditions of cultivation, growth stage, and substrate availability. In this study, diverse concentrations of Hg might affect the growth of bacteria and the production of exogenous substances, which is something that has already been observed by Shokri and Emtiazi [31]. In their characterization of IAA-producing Gram-negative bacteria, including the genera *Agrobacterium*, *Rhizobium*, *Klebsiella*, and *Azotobacter*, they found maximum yields between 4.90 μg/mL and 5.2 μg/mL, which is a range of mean values similar to those found in this study. Surprisingly, some strains produce higher values (9, 21, 31, 37, 56, 95, and 98). However, authors of such studies do not measure IAA production in the presence of specific metal and, therefore, do not take into account the possible biochemical alterations resulting from the presence of a toxic metal, which may have a negative impact on IAA production.

Bacterial siderophores are molecules secreted in conditions of iron deficiency in order to sequester metal from their environment [32]. This paper compares the production capacity of these compounds in conditions with various concentrations of Hg, in contrast to the results obtained by Robas et al. [11], and the findings show a total inhibition of siderophore production at concentrations of 80 μg/mL and higher. Other studies [33,34] have highlighted the importance of siderophores in protecting bacteria and plants from the hyper-accumulation of toxic metals. According to such studies, the production of some siderophores is induced by the presence of low concentrations of Hg in the medium (5 μg/mL), establishing analogous competition between Fe and Hg. The results described in this paper do not rule out the possibility that the bacteria tested have not produced siderophores due to the fact that there was a maximum concentration of up to 7 μg/mL of exchangeable Hg in the edaphic medium from which they were isolated [35]. 

Another PGPR strategy is to decrease ethylene levels in plants. Ethylene regulates plant growth linked to abiotic stress through the activity of the enzyme ACC deaminase (ACCd), which deaminates the immediate precursor of ethylene, the ACC [36]. ACCd levels vary widely in microorganisms since their regulation can occur at the enzymatic level or according to gene expression [37]. Studies such as those by Mendoza-Hernandez et al. [38] show that certain bacteria decrease their ACCd activity when subjected to the presence of heavy metals, which could be the case of our study herein, in which there are high concentrations of Hg.

Finally, phosphate-solubilizing microorganisms facilitate the access of plant rhizospheres to these salts, which are absorbed by the plant, improving its growth and productivity [39]. In the presence of Hg, a decrease in the number of phosphate solubilizing strains can be observed. However, those strains that retain this activity are able to solubilize phosphates even with high concentrations of Hg, making them very good candidates for later use as adjuvant bioremediation PGPRs. 

Authors such as Emami et al. [40] or Bomfim et al. [41], suggest that promoting successful growth must be linked to the diverse mechanisms that operate synergistically during plant development, not just to one of them. Successful remediation by the selected strains will be a result of the combined activities of the microorganism. Therefore, in this study, the quantification of the remediation potential of the strains has been used through the application of BMRSI [11]. 

The genera *Bacillus* and *Pseudomonas* are described as especially abundant in the composition of edaphic bacterial communities in numerous studies. This microbiota is greatly affected by seasonal factors, as well as others, since its prevalence increases in spring and autumn when the level of moisture and photosynthates is high [42,43,44]. Following the criteria established by Robas et al. [11], strains with BMRSI values ≥ 6.5 and IAA production > 5.5 µg/mL. were selected, and four of the strains met the selection criteria. 

Strain 9 has been identified as *Bacillus toyonenesis.* Recent studies have described the use of this bacterium as a PGPR through the production of IAA [45]. Likewise, other studies have been published, such as those of Naguib et al. [27], regarding its tolerance to Hg. This strain has one of the highest BMRSI values in the sample (7.30), making it a good candidate for further use in bioremediation.

Tolerance to Hg has also been reported with *Brevibacterium frigoritolerans* in a study of the microbial community in sediments of the Aussa River. Khezrinejad et al. [46] have proposed the use of this bacterium as a PGPR due to its strength in producing IAA. For this reason, Strain 25 is noteworthy, as it has a BMRSI of 6.54 and an IAA production capacity of 6.30 µg/mL. 

In 2006, in the area of Huelva, López et al. [47] isolated five strains from wedge sole (*Dicologlossa cuneata*), which were producing disease in a human adult. Among the isolated species, a new species was found, known as *Pseudomonas baetica*. Strain 98 is also worthy of mention, as it has a high tolerance to Hg (160 μg/mL) and a high level of auxin production (6.76 μg/mL), giving it the third-highest BMRSI value in the sample. This makes it one of the best candidates to be analyzed for its capability in promoting growth in model plants. 

Strain 21 was identified as *Pseudomonas moraviensis.* This species was first isolated by Tvrzova et al. [48] in an experiment involving the selective enrichment of soil with nitroaromatic compounds. Strains of this species have also been shown to have PGPR capabilities [49,50]. Strain 21 obtained the second-highest BMRSI score of the study with 7.20 points and the highest level of auxin production (7.06 μg/mL), making it one of the best candidates as well to be studied and approved for bioremediation. 

## 5. Conclusions

1. The presence of Hg in culture mediums directly affects the capability of PGPR bacteria by decreasing their effectiveness. Such bacteria are affected in the following order, from highest to lowest affected: Siderophores > Phosphate production > ACC deaminase > IAA production.
2. The Bio-Mercury Remediation Suitability Index (BMRSI) has proven to be a useful tool for evaluating strains in an integrated way based on their PGPR capabilities in the presence of Hg. MBC (Hg) > 100 μg/mL and BMRSI ≥ 6.5 are proposed as a strain selection criterion for later bioremediation of Hg-contaminated soils.
3. Based on the criteria described, the strains *Bacillus toyonensis* (9), *Pseudomonas moraviensis* (7), *Pseudomonas baetica* (26), and *Brevibacterium frigoritolerans* (95) have been selected as good candidates for further phyto-rhizoremediation trials of Hg-contaminated soils.

## Figures and Tables

**Table 1:** Strains analyzed with their corresponding BMRSI values, obtained by Robas et al. [11].

Strain	1	9	10	11	18	20	21	23	31	35	37	43	48	50
BMRSI	6.68	6.56	7.42	7.69	7.88	7.56	7.21	6.97	7.40	5.02	7.07	7.69	6.62	7.09
Strain	55	56	57	58	69-I	69-II	70	74	75	76	79	80	95	98
BMRSI	7.23	6.43	7.26	6.46	7.86	8.51	6.35	8.07	6.30	7.04	7.55	8.42	7.57	7.05
Strain	114	122	130	146	149	160	173	175	204	211	211-I	214	114	122
BMRSI	7.67	6.59	8.01	7.99	6.26	6.32	6.60	7.08	6.80	7.74	7.64	5.40	7.67	6.59

**Table 2:** Strains ordered by BMRSI descending values with all factors integrated, IAA: IAA production; PO_4_^3−^: solubilization of phosphates; ACCd: degradation of ACC via ACC deaminase; MBC: maximum bactericidal concentration. 0/1 indicates absence/presence. ND: not defined bacteria.

Strain	Identification	IAA (μg/mL)	PO_4_^3^^−^	ACCd	MBC (μg/mL)	BMRSI
9	*Bacillus toyonensis*	6.16	0	1	140	7.30
21	*Pseudomonas moraviensis*	7.06	0	0	140	7.20
98	*Pseudomonas baetica*	6.76	0	0	160	6.92
95	*Brevibacterium frigoritolerans*	6.40	0	0	140	6.54
37	*Pseudomonas fluorescens*	6.08	0	0	140	6.22
56	*Pseudomonas brassicacearum subsp. brassicacearum*	6.05	0	0	160	6.21
58	*Pseudomonas brassicacearum subsp. brassicacearum*	4.70	0	1	160	5.86
31	*Pseudomonas brassicacearum subsp. brassicacearum*	5.67	0	0	140	5.81
122	*Brevibacterium frigoritolerans*	4.37	0	1	160	5.53
50	*Bacillus toyonensis*	4.15	1	0	350	5.50
173	*Bacillus toyonensis*	3.93	0	1	180	5.11
48	*ND*	3.91	0	1	140	5.05
57	*Pseudomonas corrugata*	3.61	1	0	350	4.96
55	*Pseudomonas syringae pv. phaseolicola*	4.80	0	0	140	4.94
69-II	*Pseudomonas sp.*	3.77	1	0	160	4.93
70	*Pseudomonas corrugata*	4.51	0	0	350	4.86
69-I	*Pseudomonas syringae pv. phaseolicola*	4.67	0	0	160	4.83
43	*Bacillus toyonensis*	4.59	0	0	160	4.75
1	*Pseudomonas migulae*	4.59	0	0	140	4.73
23	*Pseudomonas moraviensis*	4.42	0	0	140	4.56
76	*ND*	4.10	0	0	140	4.24
204	*Brevibacterium frigoritolerans*	4.04	0	0	160	4.20
149	*Pseudomonas syringae pv. phaseolicola*	4.02	0	0	140	4.16
211	*Bacillus dendretensis*	3.85	0	0	200	4.05
114	*Pseudomonas syringae pv. phaseolicola*	3.80	0	0	140	3.94
75	*Pseudomonas syringae pv. phaseolicola*	3.74	0	0	160	3.90
79	*Pseudomonas syringae pv. phaseolicola*	3.66	0	0	140	3.80
74	*Xanthomonas oryzae pv. oryzae*	3.66	0	0	140	3.80
35	*Pseudomonas baetica*	3.64	0	0	140	3.78
20	*Pseudomonas fluorescens*	3.64	0	0	140	3.78
175	*ND*	3.50	0	0	140	3.64
130	*Pseudomonas corrugata*	3.47	0	0	140	3.61
18	*Bacillus toyonensis*	3.45	0	0	140	3.59
11	*Pseudomonas corrugata*	3.34	0	0	200	3.54
146	*Pseudomonas fluorescens*	3.20	0	0	180	3.38
10	*ND*	3.09	0	0	140	3.23
160	*Bacillus circulans*	3.09	0	0	140	3.23
211	*Bacillus dendretensis*	2.88	0	0	200	3.08
214	*Bacillus niacini*	2.82	0	0	200	3.02
80	*Pseudomonas syringae pv. phaseolicola*	2.85	0	0	140	2.99

## Data Availability

No new data were created or analyzed in this study. Data sharing is not applicable to this article.
